# Albuminuria, structural brain findings and Circulating biomarkers of brain injury in older adults

**DOI:** 10.1038/s41598-025-06448-1

**Published:** 2025-07-01

**Authors:** Jens W. Horn, Alison Fohner, Russell Tracy, Hieab H. H. Adams, Luc Djousse, Solfrid Romundstad, Imre Janszky, W. T. Longstreth, Kenneth J. Mukamal

**Affiliations:** 1 Department of Public Health and Nursing, Norwegian University of Science and Technology, Trondheim, Norway; 2 Department of Internal Medicine, Levanger Hospital, Health Trust Nord-Trøndelag, Levanger, Norway; 3 Department of Epidemiology, University of Washington, Seattle, WA USA; 4 Institute of Public Health Genetics, University of Washington, Seattle, WA USA; 5 Department of Biostatistics, University of Washington, Seattle, WA USA; 6 Department of Pathology & Laboratory Medicine and Biochemistry, Larner College of Medicine, University of Vermont, Burlington, USA; 7 Department of Clinical Genetics and Department of Radiology and Nuclear Medicine, Erasmus University Medical Center, Rotterdam, The Netherlands; 8 Latin American Brain Health (BrainLat), Universidad Adolfo Ibáñez, Santiago, Chile; 9 Division of Aging, Department of Medicine, Brigham and Women’s Hospital and Harvard Medical School, Boston, MA USA; 10 Department of Clinical and Molecular Medicine, Norwegian University of Science and Technology, Trondheim, Norway; 11 Department of Global Public Health, Karolinska Institutet, Stockholm, Sweden; 12 Regional Center for Health Care Improvement, St. Olav’s University Hospital, Trondheim, Norway; 13 Department of Neurology, University of Washington, Seattle, WA USA; 14 Department of Medicine, Beth Israel Deaconess Medical Center, Boston, MA USA; 15 Department of Public Health and Nursing, Norwegian University of Science and Technology, Forskningsvegen 2, 7600 Levanger, Norway

**Keywords:** Epidemiology, Risk factors, Magnetic resonance image, Albuminuria, Cerebral small vessel disease, Neurofilament light chain, Neurology, Risk factors

## Abstract

Albuminuria reflects systemic endothelial dysfunction, but its relationships with subclinical brain abnormalities have not been comprehensively catalogued. The Cardiovascular Health Study recruited older adults from four US communities, beginning in 1989–1990. Systematic measurements of albuminuria were performed in 1996–1997; two brain MRIs, in 1992–1994 and 1997–1999; and serum neurofilament light chain (NfL) measurements from 1996–1997 stored samples. We examined the associations of albuminuria with longitudinal progression of white matter hyperintensities (WMH) and ventricular size, incident infarcts, and cross-sectional quantitative brain volumes and circulating biomarkers of neuronal injury (n = 834–1950). Albuminuria was positively associated with ventricular grade worsening (odds ratio per doubling 1.10, 95% confidence interval (CI) 1.01–1.19) and with circulating NfL levels (2% higher per doubling, 95% CI 1–4%), even after adjustment for vascular risk factors. Albuminuria was also associated with worsening of WMH, incident infarcts, and quantitative WMH and hippocampal volumes, but these latter associations appeared to reflect burden of cardiovascular risk factors. Albuminuria is independently associated with worsening ventricular size and circulating NfL, suggesting a specific role of microvascular dysfunction in brain atrophy. It also reflects cardiovascular risk factor burden on markers of vascular brain injury. These results highlight the diverse associations of albuminuria with common brain abnormalities of aging.

**Supplementary Information:**

The online version contains supplementary material available at 10.1038/s41598-025-06448-1.

## Introduction

Aging is associated with several simultaneous pathophysiological processes in the brains of older adults, including progressive atrophy and cerebral small vessel disease (CSVD). These have major prognostic import; CSVD, for example, is associated with cognitive impairment, depression, incident stroke, dementia, and death^1–3^. Given the paucity of therapeutic options for patients with established dementia or previous stroke, establishing risk factors for these processes in older adults that might point to preventative therapies or better identify individuals at high risk remains an important challenge. As a result, easily measured markers of vascular dysfunction are attractive targets as risk factors for aging-related brain abnormalities.

Albumin secretion in the urine (albuminuria) and lower estimated glomerular filtration rate (eGFR) both indicate kidney failure as an independent risk factor for cardiovascular diseases and dementia, so that accounting for eGFR is important for isolating the use of albuminuria as a marker of endothelial dysfunction. As albuminuria grows more severe, however, it increasingly reflects kidney disease per se, rather than reflects endothelial dysfunction, so that exclusion of individuals with severe albuminuria may be needed^4^. However, studies of albuminuria and structural brain findings remain largely cross-sectional and inconsistent in their findings. Cross-sectional studies using magnetic resonance imaging (MRI) have linked urinary albumin-creatinine ratio (ACR) with white matter hyperintensities^4–9^ and lacunar infarcts^6–8^. Results for brain atrophy have been less consistent, with variable findings for generalized brain atrophy^5,10^, a positive association with cortical thinning^9^, and no association with ventricular^5^ or hippocampal atrophy^10^. Longitudinal studies have also yielded inconsistent results, as the AGES-Reykjavik Study showed that initially elevated or increasing ACR over a 5-year period was associated with increased white matter hyperintensities, but no association with incident infarction^11^. In a study of 360 hypertensive participants assessed 4 years apart, incident microalbuminuria at the follow-up visit was also associated with progression of periventricular white matter hyperintensity (WMH) but not with incident infarction^12^, and the association with progressive focal or global brain atrophy was ambiguous^10,13^.

In addition to MRI findings, circulating levels of CNS-derived circulating biomarkers, such as neurofilament light chain (NfL) and glial fibrillary acidic protein (GFAP), have also been associated with progression and severity of subclinical brain abnormalities^14–17^. However, their association with ACR after adjusting for estimated glomerular filtration rate (eGFR), to account for reduced kidney function and potential confounding, has yet to be evaluated.

**Fig. 1:**
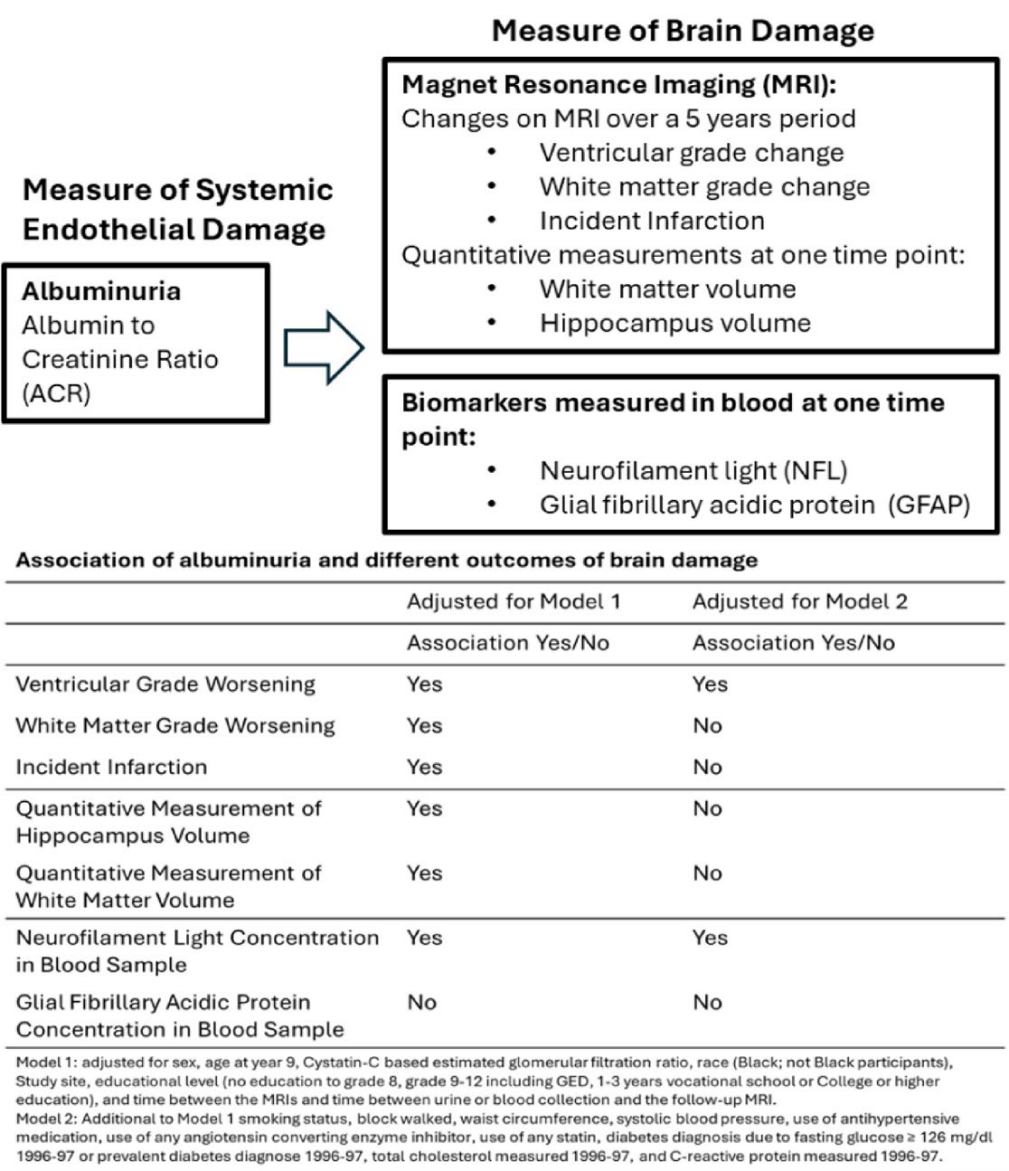
Overview of the different analysis and results.

To address these questions, we studied the comprehensive associations of ACR, as a measure of systemic endothelial damage (and not as measure of kidney function), with a broad array of measures of subclinical brain injuries in the Cardiovascular Health Study (CHS). These measures included longitudinal change in two structural brain findings by MRI and with other cross-sectional quantitative estimates of WMH and hippocampal volumes and two circulating biomarkers. CHS is an ongoing population-based cohort study of older adults that systematically ascertained ACR, NfL, and GFAP. It includes two brain MRIs conducted approximately 5 years apart, offering a unique opportunity to evaluate the associations between albuminuria and age-related brain pathophysiology, both independently and as a marker of overall vascular risk. (Fig. 1).

## Methods

### Cohort

The CHS began in 1989–1990 and recruited 5201 women and men aged 65 years and older in 4 different US communities: Forsyth County, North Carolina; Washington County, Maryland; Sacramento County, California and Pittsburgh, Pennsylvania^18^. In 1992–1993, additional 687, predominantly African American participants were recruited in 3 of the 4 original communities. Potential participants were randomly identified from Medicare eligibility lists, and exclusion criteria included active treatment for cancer, use of a wheelchair, and intent to move within 3 years. Participants were examined annually at their respective field centers until 1998–1999.

**Fig. 2:**
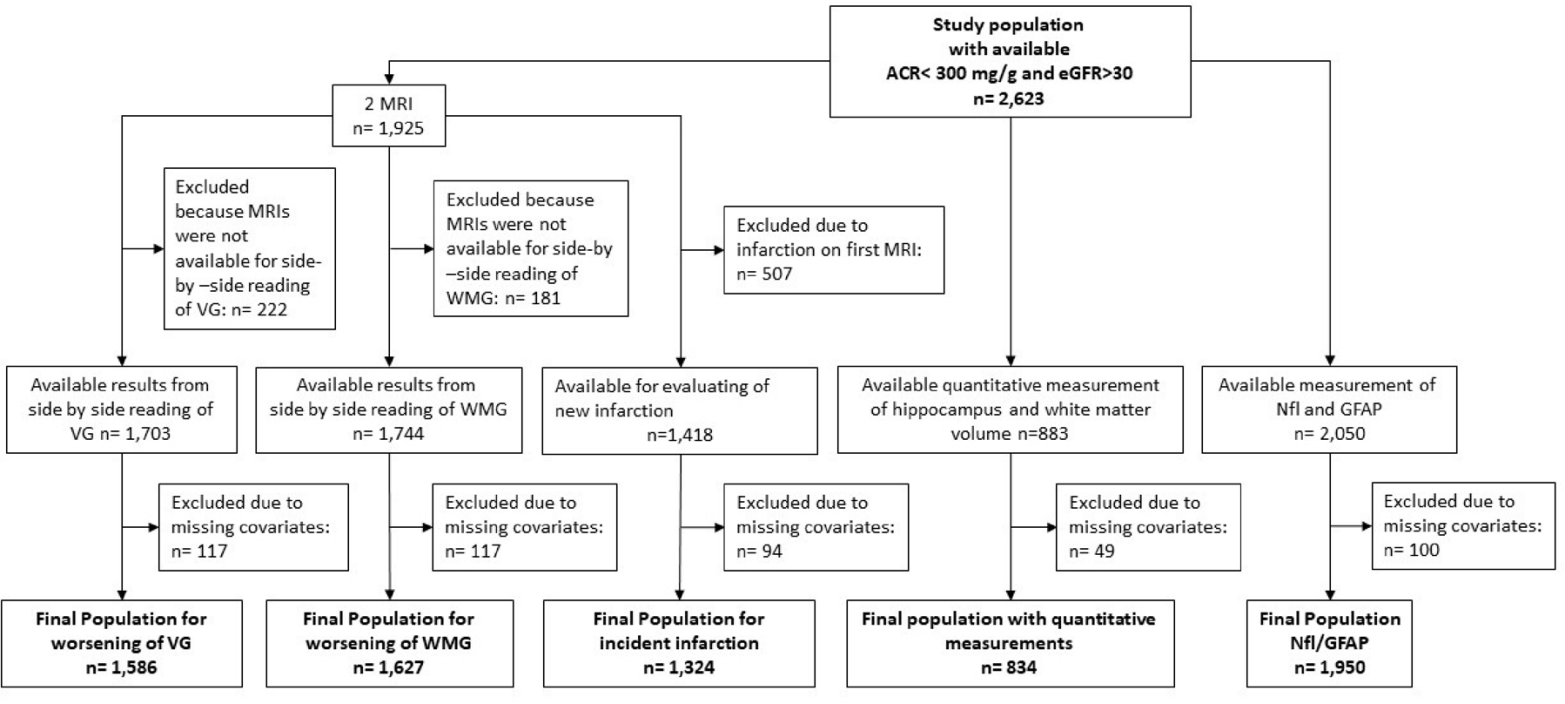
Flowchart of the study population.

For the analyses described in this report, we utilized data from several cross-sectional CHS examinations. Figure 2 shows the flow and number of participants included in each of our analyses.

### Urinary albumin-creatinine ratio (ACR)

The 1996–1997 field center examination included, among other measurements, a random spot morning urine test. Urine was sent in frozen aliquots to the CHS Central Laboratory, where urinary albumin was measured immediately after defrosting by rate nephelometry, using the Array 360 CE Protein Analyzer (Beckmann Instruments, Fullerton, CA). Urinary creatinine was measured on a Kodak Ektachem 700 Analyzer. ACR was calculated by dividing albumin in mg/dl by creatinine in mg/dl multiplied by 1000, resulting in ACR in mg/g. We excluded participants with macroalbuminuria (300 mg/g) or eGFR < 30 l/min/1.73 m^2^, which may indicate kidney failure, from primary analysis. We subsequently examined ACR as both a linear and binary variable, dichotomized at the standard cut off of moderately increased albuminuria, of 30 mg/g^19^.

### Brain imaging

We examined two sets of imaging outcomes in these analyses. First, we examined the relationship of ACR to semiquantitative longitudinal worsening in ventricular size and WMH and to incident MRI-defined infarction, estimated from MRIs conducted 5 years apart—approximately 4 years before to 1 year after the ACR assessment. Second, we evaluated quantitative volumes of the hippocampi and WMH assessed on the follow-up MRI.

Cranial MRI was offered twice to CHS participants in 1992–1994 and again in 1997–1999^20,21^. Results were available from both timepoints for 1925 participants with available ACR < 300 mg/g and eGFR > 30 l/min/1.73 m^2^, with a mean of 5 years between scans. The scanning protocol included sagittal T1-weighted localizer images and axial T1, spin-density, and T2-weighted images. Axial images had 5-mm thickness without interslice gaps^22^. The baseline scans were performed on General Electric or Picker 1.5-T scanners at three field centers and on a 0.35-T Toshiba instrument at the fourth^23^. The follow-up MRI scans were all performed on 1.5 T General Electric MRI scanners. MRI scanner specifications and scanning protocols are presented in Supplemental Table 1. The radiological images were sent to a single reading center. Neuroradiologists, blinded for the participants age, sex, race, ethnicity, and clinical information, evaluated the images, using a standardized protocol^16,20,23^.

**Table 1:** Characteristics in participants with measurement of ACR and the different outcomes.

Characteristics	Population with ACR and 2 MRI, side by side reading for worsening of VG: n = 1586	Population with ACR and 2 MRI, side by side reading for WMG: n = 1627	Population for incident stroke: N = 1324	Population with quantitative measurement of hippocampus and WM lesion volume: n = 834	Population with ACR, and measurement of Nfl and GFAP: n = 1950
Age, years	77.6 ± 4.33	77.6 ± 4.33	77.3 ± 4.18	77.7 ± 4.2	77.7 ± 4.40
Female sex	945 (59.6)	972 (59.7)	795 (60.1)	493 (59.1)	1185 (60.7)
Race, not Black	1342 (84.6)	1381 (84.9)	1125 (85.0)	732 (87.8)	1681 (86.1)
Educational status					
No education to grade 8	159 (10.0)	162 (9.9)	123 (9.3)	66 (7.9)	202 (10.4)
Grade 8–12, including GED	629 (39.7)	650 (40.0)	531 (40.1)	307 (36.8)	769 (39.4)
1–3 year vocational school	138 (8.7)	140 (8.6)	120 (9.1)	72 (8.6)	171 (8.8)
College or higher education	660 (41.6)	675 (41.5)	550 (41.5)	389 (46.6)	808 (41.4)
Waist circumference, cm	96.2 ± 12.6	96.1 ± 12.6	96.5 ± 12.8	95.7 ± 12.5	96.4 ± 12.6
Smoking status					
Never	793 (50.0)	815 (50.1)	669 (50.5)	401 (48.1)	963 (49.4)
Former	677 (42.7)	693 (42.6)	567 (42.8)	379 (45.4)	850 (43.6)
Current	116 (7.3)	119 (7.3)	88 (6.7)	54 (6.5)	137 (7.0)
Walked Blocks	43.1 ± 74.4	42.9 ± 73.7	44.6 ± 73.9	43.3 ± 67.9	40.3 ± 67.7
Systolic blood pressure, mmHg	135.9 ± 19.5	135.9 ± 19.6	135.1 ± 19.6	135.5 ± 19.9	136.3 ± 20.5
C-reactive protein mg/l	4.4 ± 7.7	4.3 ± 7.6	4.3 ± 8.0	4.3 ± 8.8	4.4 ± 7.9
Fasting total cholesterol mg/dl	202.0 ± 38.8	202.0 ± 38.7	201.2 ± 37.5	202.4 ± 38.0	202.6 ± 38.4
Cystatin C-based eGFR, l/min/1.73 m^2^	72.9 ± 18.1	72.9 ± 18.1	73.8 ± 18.0	73.5 ± 18.4	72.7 ± 17.6
Usen of anti-hyper-tensive medication	861 (54.3)	883 (54.3)	696 (52.6)	424 (50.8)	1024 (52.5)
Use of ACE Inhibitor	204 (12.9)	208 (12.8)	154 (11.6)	100 (12.0)	240 (10.4)
Use of Statins (HMG-CoA RI)	165 (10.4)	169 (10.4)	121 (9.1)	90 (10.8)	193 (9.9)
Diagnosis diabetes at baseline or increased fasting glucose ≥ 126 mg/dl	247 (15.6)	248 (15.2)	191 (14.4)	108 (13.0)	118 (6.1)
Congestive Heart Failure History	91 (5.7)	95 (5.8)	66 (5.0)	47 (5.6)	116 (6.0)
Atrial Fibrillation or Flutter	95 (6.0)	97 (6.0)	79 (6.0)	49 (5.9)	131 (6.7)
Adjudicated Stroke at study 1996–97	78 (4.9)	79 (4.9)	38 (2.9)	39 (4.7)	98 (5.0)
APO ε4, (any ε4 allele)	357 (24.5) (n = 1459)	364 (24.3) (n = 1500)	290 (23.9) (n = 1212)	188 (24.2) (n = 776)	448 (25.2) (n = 1780)
Albumin/Creatinine Ratio in urine, mg/g	18.5 ± 34.1	18.6 ± 34.4	17.7 ± 34.3	18.5 ± 33.3	17.8 (33.6)
Nfl from serum, pg/ml	30.3 ± 25.2 (n = 1132)	30.8 ± 30.3 (n = 1162)	29.7 ± 28.5 (n = 976)	30.3 ± 22.3 (n = 639)	31.1 ± 31.2 (n = 1950)
GFAP from serum, pg/ml	282.8 ± 195.4 (n = 1132)	284.4 ± 197.9 (n = 1162)	282.2 ± 206.2 (n = 976)	289.2 ± 236.2 (n = 639)	284.8 ± 178.3 (n = 1950)
Time between urine/blood collection and follow-up MRI, days	473.8 ± 133.5	474.32 ± 133.6	473.8 ± 135.2	421.8 ± 91.7 (n = 834)	472.42 ± 133.8 (n = 1371)
Time between MRIs, days	1826.0 ± 224.6	1827.4 ± 225.7	1824.4 ± 226.6	1782.4 ± 210.1 (n = 834)	1822.3 ± 227.1 (n = 1288)
MRI Findings					
Available for VG assessment	1586 (100)	1586 (97.5)	1173 (88.6)	723 (86.7)	1129 (57.9)
No VG worsening	1128 (71.1)	1128 (71.1)	842 (71.8)	531 (73.4)	802 (71.1)
1 grade VG worsening	445 (28.1)	445 (28.1)	324 (27.6)	186 (25.7)	319 (28.2)
2 grade VG worsening	13 (0.8)	13 (0.8)	7 (0.6)	6 (0.8)	8 (0.7)
Available for WMG assessment	1580 (99.6)	1627 (100)	1201 (90.7)	742 (89.0)	1162 (59.6)
No WMG worsening	1141 (72.2)	1177 (72.3)	909 (75.7)	556 (74.9)	843 (72.6)
1 grade WMG worsening	374 (23.7)	384 (23.6)	256 (21.3)	160 (21.6)	270 (23.2)
≥ 2 grade WMG worsening	65 (4.1)	66 (4.1)	36 (3.0)	26 (3.5)	49 (4.2)
No infarction on initial MRI	1173 (74.0)	1201 (73.8)	1324 (100)	604 (100)	977 (75.9)
Incident infarction on follow-up MRI	213 (18.2)	223 (18.6)	244 (18.4)	96 (15.9)	172 (17.6)
Available for quantitative measurement on follow-up MRI	723 (45.5)	742 (45.6)	603 (45.5)	834 (100)	640 (32.8)
Total hippocampal volume, mm^3^	6796.3 ± 965.2	6795.0 ± 963.2	6829.6 ± 977.0	6784.4 ± 981.7	6802.5 ± 966.9
Total Volume of abnormal WMH, mm^3^	6847.9 ± 7597.2	6819.9 ± 7523.7	5735.9 ± 6387.3	6753.1 ± 7379.4	6845.4 ± 7695.9

Values are mean ± SD or n (%). If the numbers of participants deviate from the population in the column the numbers are indicated in parentheses.

ACR, albumin to creatinine ratio from urine; APO ε4, apolipoprotein ε4; MRI, magnet resonance imaging; NfL, neurofilament light; GFAP, glia fibrillary acidic protein; eGFR, estimated glomerular filtration Rate; ACE, angiotensin converting enzyme; HMG-CoA RI, β-Hydroxy β-methylglutaryl-coenzyme A reductase inhibitors; WMG, white matter grade; VG, ventricle grade; WMH, white matter hyperintensity.

Cardiovascular Health Study, baseline 1996–1997 and information on 2 MRI’s from 1992/1994 and 1997/1999.

To assess ventricular and WMH grades (VG, WMG) over time, we used MRIs eligible for side-by-side re-reading. Because of technical problems, 197 of the original 2116 pairs of scans could not be re-read, leaving 1919 (91%) pairs^24^. Ventricular sizes were estimated from the T1-weighted axial images. The size of the lateral ventricles was estimated by the radiologist based on a referenced standard to create a VG ranging from 0 (least) to 9 (most abnormal)^3^. Inter- and intra-reader reproducibilities for VG within one grade were good. Intrareader agreement within 1 grade was 94% with a kappa of 0.89 for the VG^3^. Worsening of VG by one or more grades (yes versus no) was determined by neuroradiologists in side-by-side reading.

WMH were identified on either axial T2- weighted or spin-density images scans and graded on a similar 10-point semiquantitative WMG, ranging from 0 (least) to 9 (most abnormal). WMG had an inter-reader interclass kappa correlation coefficient of 0.76 and intrareader kappa coefficient of 0.89^23^. To determining worsening of WMG by 1 or more grades (yes versus no), neuroradiologist reread all scans side-by-side^24^. The intra-reader reliability kappa coefficient for the reads was 0.59, and inter-reader reliability kappa coefficient was 0.36, so certain scans were reviewed and adjudicated as described earlier^24^.

Brain infarcts were defined as abnormal signal intensity > 3 mm. Incident brain infarction was defined as one or more infarcts on the follow-up MRI among participants whose initial MRI was free of any infarct^21^.

A subgroup of the follow-up MRIs was performed on 1.5 T scanners and included 3D T1-weighted spoiled gradient-recall sequences^25^. We applied FreeSurfer software (suite 6.0) to these scans to estimate the quantitative volume of hippocampus, white WMH, and total intracranial space. The software reformats high resolution T1-weighted images to 1 × 1 × 1 mm^3^ voxles and generates a surface-based reconstruction of the brain. We used both total volume of hippocampi and the total volume of WMH, with adjustment for total brain volume. The volume of WMH on T1 weighted images has shown strong correlation with the WMH on fluid-attenuated inversion recovery T2 sequences^26^.

### NfL and GFAP

In addition to MRI-based outcomes, we used NfL and GFAP measured in frozen fasting, previously unthawed serum, collected from participants at the 1996–1997 clinic visit. Because the measurements were funded as part of a metabolic ancillary study to CHS, these measurements were restricted to participants who completed an oral glucose tolerance test, excluding those with known diabetes. The CHS Central Laboratory at the University of Vermont measured NfL and GFAP using the 4th generation single-molecule array (SIMOA) Human Neurology 4-PlexA (Quanterix). The inter-assay coefficients of variation were 9.3% for Nfl and 8.2% for GFAP, well below the standard thresholds of 20–30%^27^.

### Covariates

Covariates were drawn from the 1996–1997 examination, during which ACR was measured, with the exception of information on sex, race, study site, and educational status, which was obtained from the baseline examination in 1989–1990.Due to low numbers of participants who were Hispanic, Asian, or other race-ethnicities, we dichotomized participants as Black or not Black. Self-reported information on education was categorized into four groups (no education to grade 8; grades 9–12; 1–3 years of vocational school; and college or higher education). We also included time from the initial MRI and from ACR sampling to the follow-up MRI as covariates in relevant models.

Smoking in 1996–1997 was categorized into never, former, or current smoking, and number of walked blocks per week was used as continuous variable. Information on medication was retrieved from a validated medication inventory and included information on use of any antihypertensive medication (HTM), angiotensin converting enzyme (ACE)-inhibitors, and statins^28^. We defined prevalent diabetes in 1996–1997 based upon use of anti-diabetic medication or a fasting glucose ≥ 126 mg/dl. Other measured continuous covariates derived from fasting blood samples included C-reactive protein (CRP), total cholesterol, and cystatin-C to calculate estimated glomerular filtration ratio in ml/min/1.73 m^2^. Trained health technicians measured waist circumference and systolic blood pressure at the index visit.

Information on prevalent stroke and congestive heart failure was validated as previously described^29^. Prevalent atrial fibrillation was based on repeated electrocardiogram examinations at each study visit. Finally, apolipoprotein ε4 (APO ε4) allele status was ascertained among participants who provided consent for use of their genetic material. The three major allelic forms of the APO ε gene were determined by PCR amplification with specific primers, and positive controls in each batch using the Hixson and Vernier method. More detailed information of the method is described elsewhere^30^.

### Statistical analysis

Baseline characteristics were presented for continuous variables as mean (SD) and for categorical ones as numbers (percentages).

Due to its skewed distribution, ACR was transformed to the binary logarithm (log_2_ACR) for regression analyses. We assessed with logistic regression the associations with the dichotomous outcomes of VG worsening, WMG worsening, and incident infarction on follow-up MRI, using ACR as both a continuous and dichotomized variable. These analyses were initially adjusted for age, sex, race, age in 1996–1997, cystatin-C based eGFR, study site, educational status, and time between initial and follow-up MRI and time between the 1996–1997 visit and the follow-up MRI (Model 1). Next, we additionally included cardiovascular risk factors, including smoking status, number of blocks walked, waist circumference, systolic blood pressure, use of HTM, use of ACE inhibitors, use of statins, prevalent diabetes, total cholesterol, and CRP (Model 2).

In a sensitivity analysis, we further adjusted these analyses for prevalent adjudicated stroke prior to the 1996–1997 visit. We also analyzed possible interaction of APO ε4 status with ACR on VG worsening and adjusted our analyses of incident infarction for prevalent congestive heart failure and atrial fibrillation as relevant covariate. In all cases, we presented the odds ratios and 95% confidence intervals for each dichotomous outcome per doubling of ACR concentration and for ACR values above versus below the 30 mg/g threshold.

The associations of log_2_ACR with hippocampal and abnormal WMH volumes were tested using linear regression, adjusted for the same covariates as above, replacing the time variable between the two MRIs with estimated total intracranial volume and time between urine sample assessment and follow-up MRI.

We also examined NfL and GFAP in linear regression models. Due to their skew, we winsorized 10 GFAP outliers to the value of 1000 pg/ml and 9 NfL outliers to the value of 250 pg/ml and transformed their values to the natural logarithm (log_n_NfL or log_n_GFAP); as a result, we back-transformed estimates and report percentage change in these biomarkers.

To inform the degree to which ACR was confounded by specific covariates, we compared an unadjusted model with ACR alone to models which included each other covariate individually. In a second approach, we present the regression coefficients for each covariate included in the full model (Model 2).

To assess whether attrition may have biased our results, we included inverse probability-weighted adjusted regression analysis in different ways. In the first of four different approaches, the likelihood of participation as basal function of baseline covariates in both MRI examinations was calculated by logistic regression. Of the baseline population, 5791 individuals had information on sex, age at baseline, cystatin C-based eGFR, race, study location, and education level, and 1856 participated in both MRIs. Stabilized inverse probability weights were then calculated by dividing the observed participation rate by the participation probability and included in model 1. To take account for outliers, we repeated the analysis with winsorized weights. (For more information and the other approaches see Supplemental Document 1 and Supplemental Table 2) Finally, we repeated the analysis with adjusted inclusion criteria for eGFR and albuminuria and presented the results for those with eGFR ≥ 30, ≥ 45, and ≥ 60 ml/min/1.73 m^2^ with and without those with ACR > 300 mg/g in Supplementary Table 3.

**Table 2:** Association of ACR and outcomes MRI-defined, comparing initial and follow-up MRIs about 5 years apart, or volumes of hippocampus and abnormal white matter in follow-up MRI, or circulating neurobiomarkers in the Cardiovascular Health Study.

	Ventricular grade worsening n = 1586			White matter grade worsening n = 1627			Incident brain infarction n = 1324			Volume of hippocampus in mm^3^ n = 834			Volume of abnormal white matter in mm^3^ n = 834			Neurofilament light chain n = 1950			Glial fibrillary acidic protein n = 1950		
	OR	95% CI	2-sided *P*	OR	95% CI	2-sided *P*	OR	95% CI	2-sided *P*	β	95% CI	2-sided *P*	β	95% CI	2-sided *P*	Stron-ger	95%CI	2-sided *P*	Stron- ger	95%CI	2-sided *P*
Log_2_ ACR (M1)	1.11	1.03–1.20	0.01	1.08	1.00–1.16	0.04	1.12	1.02–1.23	0.02	− 39	− 78–0	0.05	431	109–754	0.01	3%	2–4%	< 0.001	1%	0–2%	0.21
Log_2_ ACR (M2)	1.10	1.01–1.19	0.02	1.04	0.96–1.13	0.31	1.05	0.95–1.16	0.34	− 23	− 64–18	0.27	286	− 51–624	0.10	2%	1–4%	< 0.001	1%	0–2%	0.22
ACR ≥ 30 mg/g (M1)	1.43	1.04–1.98	0.03	1.31	0.95–1.81	0.10	1.46	0.96–2.21	0.07	− 105	− 277–67	0.23	1930	514–3346	0.01	12%	6–19%	< 0.001	5%	− 1–11%	0.10
ACR ≥ 30 mg/g (M2)	1.34	0.96–1.88	0.08	1.16	0.83–1.62	0.40	1.17	0.75–1.81	0.49	− 25	− 203–154	0.79	1389	− 87–2864	0.07	9%	3–16%	0.003	5%	− 1–11%	0.12

ACR, albumin to creatinine ratio; CI, confidence interval; OR, odds ratio; WMG, white matter grade; M1, model 1; M2, model 2.

Model 1: adjusted for sex, age at year 9, Cystatin-C based estimated glomerular filtration ratio, race (Black; not Black participants), Study site, educational level (no education to grade 8, grade 9–12 including GED, 1–3 years vocational school or College or higher education), and time between the MRIs and time between urine or blood collection and the follow-up MRI.

Model 2: Additional to Model 1 smoking status, block walked, waist circumference, systolic blood pressure, use of antihypertensive medication, use of any angiotensin converting enzyme inhibitor, use of any statin, diabetes diagnosis due to fasting glucose ≥ 126 mg/dl 1996–97 or prevalent diabetes diagnose 1996–97, total cholesterol measured 1996–97, and C-reactive protein measured 1996–97.

Due to the multiple correlated endpoints, we conducted a multiple comparison correction using the Benjamini–Hochberg procedure to correct for the false discovery rate (FDR) of dependent endpoints^31^. We conducted all statistical analysis using Stata 17 and 18 (StataCorp LP, College Station, TX, USA).

### Standard protocol approvals, registrations, and patient consents

Institutional review boards at the University of Washington and at each study site approved the study. All CHS participants provided written informed consent. All methods were performed in accordance with the relevant guidelines and regulations.

## Results

A total of 3424 participants had ACR measured in 1996–1997. We excluded participants with substantial kidney dysfunction, defined as ACR > 300 mg/g or eGFR < 30 ml/min/1.73 m^2^ to avoid kidney failure as exposure, and using albuminuria as marker of endothelial dysfunction. Figure 2 shows the number of participants in each analysis, based upon the specific outcome and exclusions; these ranged from 834 for quantitative analyses of brain volumes to 1950 for circulating biomarkers.

The mean age in 1996–1997 was about 77.6 years, with about 60% women and about 14% Black participants (Table 1). The only substantial difference was in the lower prevalence of diabetes among those in circulating biomarker analyses, given the requirement that they be free of treated diabetes at the time.

### Ventricular grade (VG) and white matter grade (WMG) progression and incident infarct

As seen in Table 2, ACR tended to be similarly associated with higher odds of VG worsening, WMG worsening, and incident infarct in Model 1, with adjusted ORs of 1.08–1.12 for each doubling of ACR concentration. When we adjusted for cardiovascular risk factors in Model 2, the association remained largely unchanged for VG worsening, with a clear independent association. The clinical measure of microalbuminuria suggests a possible 30% increased risk of VG progression over a 5-year period, independent of cardiovascular risk factors However, it was substantially attenuated for WMG worsening and incident infarct, suggesting that ACR served as a proxy for cardiovascular risk factors. (Table 2) In sensitivity analyses, a higher WMG in initial MRI might impact the association of ACR and WMG worsening, but an initially higher VG value did not influence VG worsening. (Supplemental Table 3) Sensitivity analyses that excluded participants who underwent baseline assessment with a 0.35 T MRI scanner showed slight attenuation of the results for ventricular grade worsening, but not for incident infarcts. (Supplemental Table 4) Our results were similar or stronger in analyses that used inverse probability weights to deal with attrition; for example, the odds ratio for ACR and ventricular grade was 1.11 (95% CI 1.03–1.20) in primary analyses and 1.13 (95% CI 1.05–1.22) with weighting. (Supplemental Table 2).

We found no interaction for APO ε4 and ACR on VG worsening (p for interaction 0.47), and additional adjustment for prevalent adjudicated stroke in analyses of worsening of VG and WMG did not alter our results (Supplemental Table 5). In contrast, these additional variables yielded somewhat more substantial attenuation of the association of ACR with incident infarcts.

When we examined the effect of adjustment for all covariates individually, systolic blood pressure, age and eGFR had the strongest impact on the association of ACR with incident infarct. (Supplemental Table 6 and 7).

Restricting the population to higher eGFR (≥ 60 or ≥ 45 ml/min/1.73 m^2^) with or without excluding ACR > 300 mg/g did not alter our estimates for risk of VG worsening. However, it modestly attenuated the estimate for WMG worsening in the population restricted to an eGFR ≥ 60 ml/min/1.73 m^2^, indicating a possible contribution of kidney disease to this outcome (Supplemental Table 8).

### Quantitative hippocampal and WMH volumes

Estimated total hippocampal volume was lower, and the volume of WMH was higher, with increasing ACR, when adjusted for demographic covariates (Model 1 Table 2). These estimates were attenuated for both outcomes and no longer statistically significant with additional adjustment for cardiovascular risk factors, similar to our findings for WMG progression and incident infarct. (Model 2 Table 2).

### Nfl and GFAP

The association of ACR with NfL was strong and statistically significant when adjusting for demographic confounders (Model 1 Table 2). Additional adjustment for cardiovascular risk factors minimally attenuated these estimates (Model 2 Table 2). The association of ACR with GFAP was weaker and not statistically significant and was nearly unchanged when additionally adjusted for cardiovascular risk factors (Table 2). In sensitivity analyses, additional adjustment for adjudicated stroke or APO ε4 had no effect on the estimates. (Supplemental Table 9 and 10). Restricting the analysis to a population with higher eGFR with or without restriction to ACR did not change our results (Supplemental Table 8).

Given the multiple endpoints, we tested whether our Model 1 findings for the MRI and circulating biomarker endpoints were significant with FDR correction. The p-values for association of ACR and NfL, VG worsening, WM volume, or incident brain infarction all fell below the corresponding FDR thresholds. WMG worsening (p = 0.04) and hippocampal volume (p = 0.05) both modestly exceeded the FDR thresholds of p = 0.036 and p = 0.043, respectively.

## Discussion

This comprehensive cross-sectional population-based study of older adults examined the association of albuminuria, as a marker of systemic endothelial function with the broadest array of markers of brain health published to date, with several distinct findings. First, we observed positive associations of ACR with longitudinal progression in brain atrophy, as assessed by ventricular size, and with circulating levels of NfL, a sensitive marker of neuronal injury. These associations remained significant even after extensive adjustment for vascular risk factors, establishing the importance of ACR in global brain atrophy. Second, we observed positive associations with longitudinal worsening of WMH, with incident infarcts, and with quantitative volume of WMH. These latter associations with lesions of vascular etiology were all attenuated by adjustment for cardiovascular risk factors (and particularly by age and hypertension), suggesting that ACR may serve as a useful proxy for vascular risk for these outcomes but may not be an independent risk factor.

The findings of our study extend earlier studies that observed higher risk for incident infarction among individuals with increased ACR that appeared to reflect common cardiovascular risk factors, especially hypertension^11,12^, results that largely concur with ours. The AGES-Reykjavik study^11^ and a study of hypertensive individuals with abnormal findings on their initial MRI^12^ both showed incident albuminuria to relate to WMH at follow-up, but the time course of when albuminuria developed in relation to brain findings was uncertain.

The finding of an independent association of higher ACR with general brain atrophy, as reflected in VG, suggests that microvascular disease may adversely impact neuronal longevity even beyond standard cardiovascular risk factors and CRP, although inflammation has been proposed as one pathophysiological pathway linking ACR to brain atrophy^11^. Perhaps surprisingly, we did not observe a comparably independent association with hippocampal volume, but the latter measurements were limited by the absence of quantitative measurements prior to ACR assessment and a smaller sample size. A previous study of hypertensive participants without prevalent stroke did not show an independent association between increased ACR and ventricular volume when adjusted for severity of hypertension^5^. Another study of diabetic participants did not show increased risk for reduced brain volume at baseline or at follow-up among participants with albuminuria^13^. Compared to those studies, the CHS population was more representative of general older adults and may be more generalizable.

We also assessed two biomarkers of ongoing brain damage, NfL and GFAP, that to our knowledge, have not previously been related to ACR. Circulating NfL is a nonspecific biomarker for neuroaxonal damage in diverse neurological disorders^14,17,32,33^, and recent work in CHS and elsewhere has linked NfL to WMG progression^16^ and to WMH and lacunar lesions on MRI^17^. In contrast, GFAP is a circulating biomarker of astrocyte damage, which plays a central role in blood–brain-barrier function, normal function of synapses and contributes to axonal metabolic maintenance^15^. Abnormal values of GFAP have also been associated with other neurological disorders such as traumatic brain injuries and cerebral hemorrhage^15^. In our study, we observed a weaker association between ACR and GFAP. The reason for the differences in our findings for NfL and GFAP is uncertain, but we previously demonstrated that NfL tended to be more strongly associated with vascular brain injuries than is GFAP in CHS, and the current results concord with that observation^16,34^.

The study reinforces the hypothesis that microalbuminuria could serve as an independent and integrated clinical surrogate marker of systemic endothelial damage, indicating an increased risk for degenerative brain diseases^35^.

Our study has several strengths. The study included a large population with systematically measured ACR and two MRIs performed 5 years apart, enabling longitudinal analysis of brain findings. We adjusted our models comprehensively for possible confounders. We also included multiple complementary measures of brain injury, some of which have not been previously examined in relation to ACR, providing a global overview of its associations with both abnormalities of vascular origin and global atrophy.

At the same time, CHS has important limitations. We were limited to a single measurement of ACR, precluding our ability to study its dynamic change. Our study design included a single measurement of ACR that occurred between the two study MRIs, which precludes a definitive answer to the question of temporality. While reverse causality appears less likely because MRI or biomarker differences are unlikely to result in changes in ACR (as opposed to ACR leading to brain changes), we cannot rule out this possibility with our design. Participants who underwent MRI tended to be healthier than those who declined, potentially leading to a subset in which the association of ACR with pathological MRI findings would be underestimated^24^. Reassuringly, inverse-probability weighted sensitivity analyses confirmed our main findings. Participants with known diabetes were excluded from measurements of NfL and GFAP, which limits the generalizability of those results but reduces potential confounding by diabetic polyneuropathy^33^. We cannot exclude the possibility that NfL and GFAP were minimally degraded during storage at − 70 °C^36,37^ and contributed to random variability in the samples. This would lead to a possible underestimation of the association in our study^38^. The MRIs in CHS were performed in the 1990s and did not have comparable technical performance to those used today. At one study site, a 0.35 T scanner was used at baseline, whereas 1.5 T scanners were used at all other study sites for both the initial and follow-up assessments. Baseline and follow-up scans were performed using different scanners and sequence parameters within each site. A sensitivity analysis showed a slight attenuation in some but not all outcomes after excluding participants with 0.35 T MRI at baseline, suggesting that this may have introduced variability into our findings. As with our MRI scanners, we used the most recently available FreeSurfer software available at the time of re-analysis (suite 6.0), but more recent versions (7.0+) have now been released. More recent approaches, such as automated volumetric measures of the brain, ventricles, or brain lesions, are more precise than our method of grading on an ordinal scale. We evaluated multiple correlated endpoints, with generally concordant results that were largely robust to false discovery rate correction, but we cannot exclude the possibility of false findings of association.

In this comprehensive study of older adults, albuminuria, as a marker of endothelial dysfunction, was independently associated with global brain atrophy, as reflected in longitudinal progression in VG and with higher levels of circulating Nfl, even after adjustment. It was also associated with WMG worsening, incident infarcts and quantitative volume of WMH via its association with vascular risk factors. Confirmation of these results in other studies with repeated measurements of ACR, Nfl, and MRI is necessary to confirm ACR as a risk factor for, and biomarker of, subclinical markers of brain health.

## Electronic supplementary material

Below is the link to the electronic supplementary material.

Supplementary Material 1

## Data Availability

The data used for this study are available with approved data distribution agreements from the Cardiovascular Health Study Coordinating Center at chs-nhlbi.org.
