# Supplementary material for: Albuminuria, structural brain findings and Circulating biomarkers of brain injury in older adults
Source: Sci Rep. 2025 Jul 1;15:22172. doi: 10.1038/s41598-025-06448-1 (PMC12217312; doi:10.1038/s41598-025-06448-1)
Supplement: Supplementary file 1 — Supplementary Material 1 [file 41598_2025_6448_MOESM1_ESM.docx]

Supplemental Document 1:

Description of inverse probability-weighted adjusted regression analyses.

Because our study design used a longitudinal approach to measure change between two MRIs, 5 years apart, restriction to the subpopulation that completed both MRIs may yielded biased results.

To address this issue, we used weighted sensitivity analysis with inverse-probability (IPW) of attrition.

We calculated the likelihood of participation in both MRI assessments as the outcome in a logistic regression model; the analytic sample for this model included the full CHS population at baseline with available information on selected covariates that we used in other analyses.

Information on sex, age at baseline, cystatin C-based eGFR, race, study location, and educational level was available in 5,791 individuals. Of these, 1,856 participated in both MRIs to assess ventricular change.

The predicted likelihood of participation (p) was then included in the formula to calculate the stabilized inverse probability weight (w):

w_i_= 1856/5791/p_i_

Finally, this weight was included in the logistic regression analyses of ACR and change of ventricular grade by the command [pweight=w]. (Supplemental Table 1)

In additional analysis we winsorized the weights to take account for outliers

In similar way we calculated stabilized IPW analysis with the population measured albuminuria (ACR) at year 9 (n=2,632) and attrition to have participated in 2 MRIs (n=1,739). The likelihood was conditioned on albuminuria only. The analysis was repeated with winsorized weights. (Supplemental Table 1)

In a further analysis we included IPW of the ratio of attrition from ACR assessment to participation on both MRIs divided by the attrition from baseline to assessments on both MRIs and repeated the analysis with winsorized weights. (Supplemental Table 1)

And finally, we Included IPW of the ratio of attrition from ACR assessment to participation on both MRIs divided by the attrition from ACR to participate on both MRI while in the calculation of the likelihood the denominator includes beside ACR other covariates as gender, age, race, study side and educational level and repeated the analysis with winsorized weights. (Supplemental Table 1)

| Supplemental Table 1: MRI Scanner Specifications at Baseline and Follow-Up Across Cardiovascular Health Study Field Centers | | | | |
| --- | --- | --- | --- | --- |
|  | Hopkins, Washington County, Maryland | Bowman Gray, Forsyth County, North Carolina | Davis, Sacramento County, California | Pittsburg, Pensivania |
| **First Cranial MRI 1992-94** | Toshiba 0.35T | GE Sigma1.5T | Picker 1.5T | GE Signa 1.5T |
| Scanning protocol |  |  |  |  |
| Sagittal T1 weighted | Using AC/PC line; TR 500ms, TE 20ms, 5mm, gap 0, 128x256 matrix, | Using AC/PC line; TR 500ms, TE 20ms, 5mm, gap 0, 128x256 matrix, | Using AC/PC line; TR 500ms, TE 20ms, 5mm, gap 0, 128x256 matrix, | Using AC/PC line; TR 500ms, TE 20ms, 5mm, gap 0, 128x256 matrix, |
| Angled axial spin density/T2-weighted | TR 3000ms, TE30/100, 5mm, gap 0, 256x192 matrix; 1nex | TR 3000ms, TE30/100, 5mm, gap 0, 256x192 matrix; ½ nex | TR 3000ms, TE30/100, 5mm, gap 0, 256x192 matrix; ½ nex | TR 3000ms, TE30/100, 5mm, gap 0, 256x192 matrix; ½ nex |
| Angled axial T1-weighted | TR 500ms, TE 20 ms, 5mm, gap 0, 256x192 matrix, 2 nex | TR 500ms, TE 20 ms, 5mm, gap 0, 256x192 matrix, 1 nex | TR 500ms, TE 20 ms, 5mm, gap 0, 256x192 matrix, 1 nex | TR 500ms, TE 20 ms, 5mm, gap 0, 256x192 matrix, 1 nex |
| **Second Cranial MRI 1997-99** | GE Signa 1.5T | GE Signa Advantage 1.5T | GE Signa Advantage 1.5T | GE Signa Advantage 1.5T |
| Scanning protocol |  |  |  |  |
| Sagittal T1Weighted | Using AC/PC line, TR 500(400-600), TE20 (<35), 5mm, 0 gap, 256x128 matrix 1 nex, FOV 24cm | Using AC/PC line, TR 500(400-600), TE20 (<35), 5mm, 0 gap, 256x128 matrix 1 nex, FOV 24cm | Using AC/PC line, TR 500(400-600), TE20 (<35), 5mm, 0 gap, 256x128 matrix 1 nex, FOV 24cm | Using AC/PC line, TR 500(400-600), TE20 (<35), 5mm, 0 gap, 256x128 matrix 1 nex, FOV 24cm |
| Angled axial spin density SD/T2 weighted | TR 3000 (>2500), TE 30/100 (<35/>75), flow comp, 5mm, interleaved, 256x192 matrix, ½ nex, variable band width (SD=16 kHz/T2=4), FOV 24 cm | TR 3000 (>2500), TE 30/100 (<35/>75), flow comp, 5mm, interleaved, 256x192 matrix, ½ nex, variable band width (SD=16 kHz/T2=4), FOV 24 cm | TR 3000 (>2500), TE 30/100 (<35/>75), flow comp, 5mm, interleaved, 256x192 matrix, ½ nex, variable band width (SD=16 kHz/T2=4), FOV 24 cm | TR 3000 (>2500), TE 30/100 (<35/>75), flow comp, 5mm, interleaved, 256x192 matrix, ½ nex, variable band width (SD=16 kHz/T2=4), FOV 24 cm |
| Angled axial T1-weighetd | TR 500 (400-600), TE 20 (<35), 5mm interleaved, 256x192 matrix, 1 nex, ¾ rectangular FOV 24cm | TR 500 (400-600), TE 20 (<35), 5mm interleaved, 256x192 matrix, 1 nex, ¾ rectangular FOV 24cm | TR 500 (400-600), TE 20 (<35), 5mm interleaved, 256x192 matrix, 1 nex, ¾ rectangular FOV 24cm | TR 500 (400-600), TE 20 (<35), 5mm interleaved, 256x192 matrix, 1 nex, ¾ rectangular FOV 24cm |
| Axial SPGR T1 weighted | SAT inferior, graphic Rx, TE 5, TR 35, 45 degree flip angel, FOV 24 cm 1 nex, frequency direction | SAT inferior, graphic Rx, TE 5, TR 35, 45 degree flip angel, FOV 24 cm 1 nex, frequency direction | SAT inferior, graphic Rx, TE 5, TR 35, 45 degree flip angel, FOV 24 cm 1 nex, frequency direction | SAT inferior, graphic Rx, TE 5, TR 35, 45 degree flip angel, FOV 24 cm 1 nex, frequency direction |

| Supplemental Table 2: Association of ACR and ventricular grade worsening on MRI, comparing initial and follow-up MRIs about 5 years apart, Cardiovascular Health Study, baseline 1996-1997 and information on 2 MRI`s from 1992/1994 and 1997/1999. Analyzes compared to sensitivity analyses additional adjusted for different invers probability ratios (IPW) of attrition to participation to the study. | | | | |
| --- | --- | --- | --- | --- |
|  |  | OR | 95% CI | 2-sided *P* |
| Log_2_ACR (Model1) | From Table 2 | 1.11 | 1.03 – 1.20 | 0.01 |
| Log_2_ACR (Model1) | Including IPW of attrition from baseline to participation on both MRI assessments. | 1.13 | 1.05 – 1.22 | 0.002 |
| Log_2_ACR (Model1) | Including IPW of attrition from baseline to participation on both MRI assessments, winsorized 1% and 99% percentiles. | 1.13 | 1.05 – 1.23 | 0.002 |
| Log_2_ACR (Model1) | Including IPW of attrition from ACR assessment to participation on both MRI assessments. | 1.11 | 1.03 – 1.20 | 0.005 |
| Log_2_ACR (Model1) | Including IPW of attrition from ACR assessment to participation on both MRI assessments, winsorized 1% and 99% percentiles. | 1.11 | 1.03 – 1.20 | 0.005 |
| Log_2_ACR (Model1) | Including IPW of the ratio of attrition from ACR assessment to participation on both MRIs divided by the attrition from baseline to assessments on both MRIs. | 1.12 | 1.04 – 1.20 | 0.003 |
| Log_2_ACR (Model1) | Including IPW of the ratio of attrition from ACR assessment to participation on both MRIs divided by the attrition from baseline to assessments on both MRIs, winsorized the weight to 1% and 99% percentiles before calculating the ratio. | 1.12 | 1.04 – 1.20 | 0.004 |
| Log_2_ACR (Model1) | Including IPW of the ratio of attrition from ACR assessment to participation on both MRIs divided by the attrition from ACR to participate on both MRI while in the calculation of the likelihood the denominator includes beside ACR other covariates as gender, age, race, study side, educational level. | 1.11 | 1.03 – 1.19 | 0.006 |
| Log_2_ACR (Model1) | Including winsorized IPW of the ratio of attrition from ACR assessment to participation on both MRIs divided by the attrition from ACR to participate on both MRI while in the calculation of the likelihood the denominator includes beside ACR other covariates. | 1.11 | 1.03 – 1.19 | 0.006 |
| ACR= Albumin to Creatinine Ratio; CI= Confidence interval; OR= Odds Ratio.  Model 1: adjusted for sex, age at year 9, Cystatin-C based estimated glomerular filtration ratio, race, Study site, educational level, and time between the MRIs and time between urine or blood collection and the follow-up MRI. | | | | |

| Supplemental Table 3. Associations of Log_2_ACR (continuous) with MRI Phenotypes according to Baseline Values on the first MRI | | | |
| --- | --- | --- | --- |
| Ventricular Grade (VG) | OR | 95% CI | 2-sided *P* |
| Model 1 if VG<4 on first MRI (n=1001) | 1.11 | 1.00-1.22 | 0.04 |
| Model 1 if VG≥4 on first MRI (n=585) | 1.13 | 1.00-1.28 | 0.05 |
| Model 2 if VG<4 on first MRI | 1.11 | 1.00-1.23 | 0.05 |
| Model 2 if VG≥4 on first MRI | 1.07 | 0.94-1.23 | 0.31 |
| White Matter Grade (WMG) |  |  |  |
| Model 1 if WMG<2 on first MRI (n=816) | 1.06 | 0.94-1.19 | 0.35 |
| Model 1 if WMG≥2 on first MRI (n=811) | 1.06 | 0.96-1.17 | 0.22 |
| Model 2 if WMG<2 on first MRI | 1.00 | 0.88-1.14 | 0.99 |
| Model 2 if WMG≥2 on first MRI | 1.04 | 0.94-1.16 | 0.43 |
| ACR= Albumin to Creatinine Ratio; CI= Confidence interval; OR= Odds Ratio; WMG= White Matter Grade.  Model 1: adjusted for sex, age at year 9, Cystatin-C based estimated glomerular filtration ratio, race (Black; not Black participants), Study site, educational level (no education to grade 8, grade 9-12 including GED, 1-3 years vocational school or College or higher education), and time between the MRIs and time between urine or blood collection and the follow-up MRI.  Model 2: Additional to Model 1 smoking status, block walked, waist circumference, systolic blood pressure, use of antihypertensive medication, use of any angiotensin converting enzyme inhibitor, use of any statin, diabetes diagnosis due to fasting glucose ≥ 126 mg/dl 1996-97 or prevalent diabetes diagnose 1996-97, total cholesterol measured 1996-97, and C-reactive protein measured 1996-97. | | | |

| Supplemental Table 4. Sensitivity analysis of the association between ACR and MRI-defined outcomes. Comparison of initial and follow-up MRIs approximately 5 years apart, excluding participants with 0.35T MRI at baseline. Cardiovascular Health Study (Baseline: 1996–1997; MRI data: 1992/1994 and 1997/1999). | | | | | | | | | | | | | | | | | | |
| --- | --- | --- | --- | --- | --- | --- | --- | --- | --- | --- | --- | --- | --- | --- | --- | --- | --- | --- |
|  | Ventricular Grade Worsening | | | | | | White Matter Grade Worsening | | | | | | Incident Brain Infarction | | | | | |
|  | Original population  n= 1,586 | | | Excluded those with 0.35T MRI at baseline  n= 1,265 | | | Original population  n= 1,627 | | | Excluded those with 0.35T MRI at baseline  n= 1,292 | | | Original population  n=1,324 | | | Excluded those with 0.35T MRI at baseline  n=1,046 | | |
|  | OR | 95% CI | 2-sided *P* | OR | 95% CI | 2-sided *P* | OR | 95% CI | 2-sided *P* | OR | 95% CI | 2-sided *P* | OR | 95%CI | 2-sided *P* | OR | 95% CI | 2-sided *P* |
| Log_2_ACR (Model 2) | 1.10 | 1.01 - 1.19 | 0.02 | 1.08 | 0.99-1.18 | 0.09 | 1.04 | 0.96 - 1.13 | 0.31 | 1.01 | 0.93-1.11 | 0.76 | 1.05 | 0.95 - 1.16 | 0.34 | 1.05 | 0.93-1.18 | 0.42 |
| ACR≥30 mg/g (Model 2) | 1.34 | 0.96 - 1.88 | 0.08 | 1.29 | 0.88-1.91 | 0.20 | 1.16 | 0.83 - 1.62 | 0.40 | 0.98 | 0.66-1.45 | 0.94 | 1.17 | 0.75 - 1.81 | 0.49 | 1.15 | 0.68-1.93 | 0.60 |
| ACR= Albumin to Creatinine Ratio; CI= Confidence interval; OR= Odds Ratio; WMG= White Matter Grade.  Model 2: Adjusted for sex, age at year 9, Cystatin-C based estimated glomerular filtration ratio, race (Black; not Black participants), Study site, educational level (no education to grade 8, grade 9-12 including GED, 1-3 years vocational school or College or higher education), time between the MRIs, time between urine or blood collection and the follow-up MRI, smoking status, block walked, waist circumference, systolic blood pressure, use of antihypertensive medication, use of any angiotensin converting enzyme inhibitor, use of any statin, diabetes diagnosis due to fasting glucose ≥ 126 mg/dl 1996-97 or prevalent diabetes diagnose 1996-97, total cholesterol measured 1996-97, and C-reactive protein measured 1996-97. | | | | | | | | | | | | | | | | | | |

| Supplemental Table 5. Sensitivity analyzes of the association of ACR with MRI-defined outcomes comparing initial and follow-up MRIs about 5 years apart and additional adjustment for earlier adjudicated stroke or atrial fibrillation and congestive heart failure.  Cardiovascular Health Study, baseline 1996-1997 and information on 2 MRI`s from 1992/1994 and 1997/1999. | | | | | | | | | |
| --- | --- | --- | --- | --- | --- | --- | --- | --- | --- |
|  | Ventricular Grade Worsening  n= 1,586 cases= 458 (29%) | | | White Matter Grade Worsening  n= 1,627 cases= 450 (27.7%) | | | Incident Brain Infarction  n=1,324 cases= 244 (18.4%) | | |
|  | OR | 95% CI | 2-sided *P* | OR | 95% CI | 2-sided *P* | OR | 95%CI | 2-sided *P* |
| Log_2_ACR (Model 2) | 1.10 | 1.01 - 1.19 | 0.02 | 1.04 | 0.96 - 1.13 | 0.31 | 1.05 | 0.95 - 1.16 | 0.34 |
| Log_2_ACR (Model 2) | *1.09 | 1.01 - 1.18 | 0.03 | *1.04 | 0.96 - 1.13 | 0.33 | *1.03 | 0.93 - 1.15 | 0.52 |
| Log_2_ACR (Model 2) |  |  |  |  |  |  | ‡1.03 | 0.93 - 1.15 | 0.55 |
| ACR≥30 mg/g (Model 2) | 1.34 | 0.96 - 1.88 | 0.08 | 1.16 | 0.83 - 1.62 | 0.40 | 1.17 | 0.75 - 1.81 | 0.49 |
| ACR≥30 mg/g (Model 2) | *1.31 | 0.94 - 1.84 | 0.11 | *1.15 | 0.82 - 1.61 | 0.42 | *1.06 | 0.68 - 1.68 | 0.79 |
| ACR≥30 mg/g (Model 2) |  |  |  |  |  |  | ‡1.06 | 0.68 - 1.67 | 0.79 |
| ACR= Albumin to Creatinine Ratio; CI= Confidence interval; OR= Odds Ratio; WMG= White Matter Grade.  Model 1: adjusted for sex, age at year 9, Cystatin-C based estimated glomerular filtration ratio, race (Black; not Black participants), Study site, educational level (no education to grade 8, grade 9-12 including GED, 1-3 years vocational school or College or higher education), and time between the MRIs and time between urine or blood collection and the follow-up MRI.  Model 2: Additional to Model 1 smoking status, block walked, waist circumference, systolic blood pressure, use of antihypertensive medication, use of any angiotensin converting enzyme inhibitor, use of any statin, diabetes diagnosis due to fasting glucose ≥ 126 mg/dl 1996-97 or prevalent diabetes diagnose 1996-97, total cholesterol measured 1996-97, and C-reactive protein measured 1996-97.  * Additional adjusted for adjudicated stroke 1996-97.  ‡ Additional adjusted for atrial fibrillation and congestive heart failure. | | | | | | | | | |

| Supplemental Table 6. Change in correlation coefficient (β) from an unadjusted model by adding a single covariate in a logistic regression analyzes of log albumin to creatinine ratio (continuous variable) and MRI-defined outcomes comparing initial and follow-up MRIs about 5 years apart.  Cardiovascular Health Study, baseline 1996-1997 and information on 2 MRI`s from 1992/1994 and 1997/1999. | | | | | | | | |
| --- | --- | --- | --- | --- | --- | --- | --- | --- |
|  | |  | Ventricular Grade Worsening  n= 1,586 | | White Matter Grade Worsening  n= 1,627 | | Incident infarction on MRI2  n= 1,324 | |
|  | |  | β | % Change | β | % Change | β | % Change |
| Crude analyzes without adjustment | |  | 0.112635 | Ref | 0.074355 | Ref | 0.1204502 | Ref |
| Hypertension (sys BP and HTN med) | |  | 0.1058062 | 6% decreased | 0.0575446 | 23% decreased | 0.0709337 | 41% decreased |
| Diabetes | |  | 0.1115828 | 1% decreased | 0.0743095 | 1% decreased | 0.1197327 | 1% decreased |
| Estimated glomerular filtration ratio | |  | 0.1082547 | 12% decreased | 0.0702878 | 5% decreased | 0.1119264 | 7% decreased |
| Use of ACE inhibitor medication | |  | 0.1095194 | 11% decreased | 0.0768775 | 3% increased | 0.1182749 | 2% decreased |
| Age | |  | 0.0986114 | 20% decreased | 0.0686738 | 8% decreased | 0 1094588 | 9% decreased |
| Sex | |  | 0.1126736 | 0% increased | 0.0757997 | 2% increased | 0.1207756 | 0% |
| Race | |  | 0.1147398 | 6% decreased | 0.0746052 | 0% | 0.1217829 | 1% increased |
| Clinic | |  | 0.1162711 | 5% decreased | 0.0819355 | 10% increased | 0.1225347 | 2% increased |
| Educational status | |  | 0.113771 | 1% increased | 0.0788145 | 6% increased | 0.1275164 | 6% increased |
| Time between MRI1 and MRI2 | |  | 0.1103168 | 2% decreased | 0.0681065 | 8% decreased | 0.1175686 | 2% decreased |
| Waist circumference | |  | 0.1120963 | 9% decreased | 0.0731726 | 2% decreased | 0.1204017 | 0% |
| Block walked | |  | 0.1125003 | 0% | 0.0714567 | 4% decreased | 0.115289 | 4% decreased |
| Smoking habit | |  | 0.1124559 | 0% | 0.0733791 | 1% decreased | 0.1200055 | 0% |
| Using HMG-CoA medication | |  | 0.111614 | 1 % decreased | 0.0738257 | 1% decreased | 0.120407 | 0% |
| Fasting total cholesterol | |  | 0.1127543 | 0% | 0.0729215 | 2% decreased | 0.1198682 | 0% |
| C-reactive protein | |  | 0.1136249 | 1% increased | 0.074906 | 1% increased | 0.120098 | 0% |
|  | |  |  |  |  |  |  |  |
| Adjudicated earlier Stroke | |  | 0.10553 | 6% decreased | 0.0715484 | 4% decreased | 0.1015683 | 16% decreased |
| Atrial Fibrillation | |  |  |  |  |  | 0.106324 | 12% decreased |
| Congestive Heart failure | |  |  |  |  |  | 0.1156764 | 4% decreased |
| APO ε4 | |  | 0.1170006 | 4% increased |  |  |  |  |
|  | β = estimated correlation coefficient; N= number of participants; APO ε4 = Apolipoprotein ε4status; *Limited measurements for APO ε4, n= 1,459; | | | | | | | |

| Supplemental Table 7. Regression coefficients for each covariate in Model 2 for MRI-defined outcomes comparing initial and follow-up MRIs about 5 years apart.  Cardiovascular Health Study, baseline 1996-1997 and information on 2 MRI`s from 1992/1994 and 1997/1999. | | | | | | |
| --- | --- | --- | --- | --- | --- | --- |
|  | Ventricular Grade Worsening  n= 1,586 | | White Matter Grade Worsening  n= 1,627 | | Incident infarction on MRI2  n= 1,324 | |
|  | β | SE | β | SE | β | SE |
| Log ACR | 0. 092517 | 0.0409 | 0.041068 | 0.0408 | 0.050199 | 0.0521 |
| Systolic blood pressure | 0.001353 | 0.0031 | 0.007150 | 0.0031 | 0.016569 | 0.0039 |
| Use hypertension medication | 0.011205 | 0.1292 | 0.081095 | 0.1281 | -0.377746 | 0.1659 |
| Use of ACE inhibitor medication | 0.251479 | 0.1786 | -0.187608 | 0.1865 | 0.188749 | 0.2390 |
| Estimated glomerular filtration ratio | 0.001144 | 0.0035 | -0.001740 | 0.0035 | -0.004548 | 0.0045 |
| Diabetes | 0.080719 | 0.1627 | 0.089716 | 0.1652 | 0.161778 | 0.2145 |
| Age at baseline | 0.042816 | 0.0142 | 0.009823 | 0.0144 | 0.017901 | 0.0187 |
| Male Sex | -0.084940 | 0.1313 | -0.212495 | 0.1320 | 0.009909 | 0.1668 |
| Black Race | -0.303935 | 0.1798 | -0.137517 | 0.1732 | -0.195280 | 0.2368 |
| Clinic |  |  |  |  |  |  |
| - Davis | -0.741566 | 0.1593 | -0.490385 | 0.1633 | -0.110935 | 0.2022 |
| - Hopkins | -0.364793 | 0.1722 | -0.390581 | 0.1797 | -0.095240 | 0.2246 |
| - Pittsburgh | -0.828958 | 0.1625 | -0.071316 | 0.1627 | -0.361858 | 0.2250 |
| Educational status |  |  |  |  |  |  |
| - Grade 9-12 | 0.185261 | 0.2083 | 0.152837 | 0.2187 | 0.180352 | 0.2888 |
| - 1-3 years vocational school | 0.245180 | 0.2679 | 0.519374 | 0.2712 | 0.571165 | 0.3446 |
| - College or higher education | 0.301482 | 0.2117 | 0.389604 | 0.2204 | 0.519479 | 0.2899 |
| Time between MRI1 and MRI2 | 0.000650 | 0.0003 | 0.001196 | 0.0003 | 0.000337 | 0.0004 |
| Time between urine or blood collection and the follow-up MRI | -0.000555 | 0.0005 | 0.000215 | 0.0005 | 0.000614 | 0.0006 |
| Waist circumference | -0.000110 | 0.0049 | -0.001291 | 0.0049 | 0.001090 | 0.0061 |
| Block walked | 0.000209 | 0.0008 | -0.001199 | 0.0009 | -0.002345 | 0.0012 |
| Smoking habit | 0.155292 | 0.0949 | 0.288109 | 0.0937 | -0.027636 | 0.1248 |
| Using HMG-CoA medication | -0.279071 | 0.1991 | -0.260900 | 0.1956 | 0.124446 | 0.2557 |
| Fasting total cholesterol | 0.001261 | 0.0016 | -0.001550 | 0.0016 | -0.000766 | 0.0021 |
| C-reactive protein | -0.000342 | 0.0081 | -0.005495 | 0.0083 | 0.005645 | 0.0090 |
| β = estimated correlation coefficient; n= number of participants; SE= standard error. | | | | | | |

| Supplemental Table 8. Association of ACR <300mg/g (or unrestricted) and MRI-defined changes or Neurofilament light in a population restricted to a eGFR ≥30, ≥45 or ≥60 ml/min/1.73 m^2^. | | | | | | | |
| --- | --- | --- | --- | --- | --- | --- | --- |
|  | Ventricular Grade Worsening | | White Matter Grade Worsening | | Neurofilament light chain | | |
|  | OR | 95% CI | OR | 95% CI | increase | 95%CI | 2-sided p |
| **Restricted to eGFR≥30**  **Original definition** | n= 1,586 | | n= 1,627 | | n= 1,950 | | |
| Log_2_ACR (Model 1) | 1.11 | 1.03 - 1.20 | 1.08 | 1.00 - 1.16 | 3% | 2 - 4% | <0.001 |
| Log_2_ACR (Model 2) | 1.10 | 1.01 - 1.19 | 1.04 | 0.96 - 1.13 | 2% | 1 - 4% | <0.001 |
| **Restricted to eGFR≥45** | n= 1,499 (1,522) | | n= 1,537 (1,560) | | n= 1,847 (1,865) | | |
| Log_2_ACR (Model 1) | 1.13 (1.10) | 1.05-1.23 (1.02-1.17) | 1.09 (1.08) | 1.01-1.18 (1.01-1.16) | 2% (3%) | 1-4% (1-4%) | <0.001(<0.001) |
| Log_2_ACR (Model 2) | 1.12 (1.08) | 1.03-1.22 (1.01-1.17) | 1.05 (1.05) | 0.97-1.14 (0.97-1.13) | 2% (2%) | 1-3% (1-3%) | 0.006 (0.001) |
| **Restricted to eGFR≥60** | n= 1,226 (1,238) | | n= 1,255 (1,267) | | n= 1,518 (1,529) | | |
| Log_2_ACR (Model 1) | 1.13 (1.10) | 1.04-1.25 (1.02-1.20) | 1.06 (1.03) | 0.96-1.16 (0.95-1.12) | 2% (2%) | 1-4% (1-3%) | 0.005 (0.007) |
| Log_2_ACR (Model 2) | 1.14 (1.10) | 1.03-1.25 (1.01-1.20) | 1.01 (1.00) | 0.92-1.12 (0.91-1.09) | 2% (1%) | 0-3% (0-3%) | 0.024 (0.036) |
| ACR= Albumin to Creatinine Ratio; CI= Confidence interval; OR= Odds Ratio; WMG= White Matter Grade.  Model 1: adjusted for sex, age at year 9, Cystatin-C based estimated glomerular filtration ratio, race (Black; not Black participants), Study site, educational level (no education to grade 8, grade 9-12 including GED, 1-3 years vocational school or College or higher education), and time between the MRIs and time between urine or blood collection and the follow-up MRI.  Model 2: Additional to Model 1 smoking status, block walked, waist circumference, systolic blood pressure, use of antihypertensive medication, use of any angiotensin converting enzyme inhibitor, use of any statin, diabetes diagnosis due to fasting glucose ≥ 126 mg/dl 1996-97 or prevalent diabetes diagnose 1996-97, total cholesterol measured 1996-97, and C-reactive protein measured 1996-97. | | | | | | | |

| Supplemental Table 9. Cross-sectional associations between ACR and circulating neurobiomarkers in the Cardiovascular Health Study population, 1996-1997, additional adjusted for adjudicated stroke. | | | | | | |
| --- | --- | --- | --- | --- | --- | --- |
|  | Neurofilament light chain  n= 1,950 | | | Glial Fibrillary Acidic Protein  n= 1,940 | | |
|  | stronger | 95%CI | 2-sided *P* | stronger | 95%CI | 2-sided *P* |
| Log_2_ACR mg/g  (Model 2) | 3% | 1 - 4% | <0.001 | 1% | 0 - 2% | 0.19 |
| Log_2_ACR mg/g  (Model 2 and adjudicated stroke) | 3% | 1 - 4% | <0.001 | 1% | 0 - 2% | 0.23 |
| ACR≥30 mg/g  (Model 2) | 9% | 3 - 16% | 0.003 | 6% | 0 - 12% | 0.06 |
| ACR≥30 mg/g  (Model 2 and adjudicated stroke) | 9% | 2 - 15% | 0.006 | 5% | 0 - 11% | 0.07 |
| ACR= Albumin to Creatinine Ratio; CI= Confidence interval.  See footnote to Table 2 for description of the models, except for excluding of the time variables time between the MRIs and time between urine or blood collection and the follow-up MRI. | | | | | | |

| Supplemental Table 10. Cross-sectional associations between ACR and circulating neurobiomarkers in the Cardiovascular Health Study population, 1996-1997, additionally adjusted for apolipoprotein ε4 status. | | | | | | |  |
| --- | --- | --- | --- | --- | --- | --- | --- |
|  | Neurofilament light chain  n= 1,780 | | | Glial Fibrillary Acidic Protein  n= 1,770 | | |  |
|  | stronger | 95%CI | 2-sided *P* | stronger | 95%CI | 2-sided *P* |  |
| Log_2_ACR mg/g  (Model 2) | 3% | 1 - 4% | <0.001 | 1% | 0 - 2% | 0.09 |  |
| Log_2_ACR mg/g  (Model 2 and apolipoprotein ε4) | 3% | 1 - 4% | <0.001 | 1% | 0 - 2% | 0.09 |  |
| ACR≥30 mg/g  (Model 2) | | 9% | 3 - 16% | 0.005 | 6% | 0 - 12% | 0.06 |
| ACR≥30 mg/g  (Model 2 and apolipoprotein ε4) | | 9% | 2 - 16% | 0.007 | 5% | 0 - 11% | 0.09 |
| ACR= Albumin to Creatinine Ratio; CI= Confidence interval.  See footnote to Table 2 for description of the models, except for excluding of the time variables time between the MRIs and time between urine or blood collection and the follow-up MRI. | | | | | | |  |
